# Relevant Word Order Vectorization for Improved Natural Language Processing in Electronic Health Records

**DOI:** 10.1038/s41598-019-45705-y

**Published:** 2019-06-25

**Authors:** Jeffrey Thompson, Jinxiang Hu, Dinesh Pal Mudaranthakam, David Streeter, Lisa Neums, Michele Park, Devin C. Koestler, Byron Gajewski, Roy Jensen, Matthew S. Mayo

**Affiliations:** 1 Department of Biostatistics & Data Science, University of Kansas Medical Center, Kansas City, KS USA; 2 University of Kansas Cancer Center, Kansas City, KS USA

**Keywords:** Data mining, Breast cancer

## Abstract

Electronic health records (EHR) represent a rich resource for conducting observational studies, supporting clinical trials, and more. However, much of the data contains unstructured text, presenting an obstacle to automated extraction. Natural language processing (NLP) can structure and learn from text, but NLP algorithms were not designed for the unique characteristics of EHR. Here, we propose Relevant Word Order Vectorization (RWOV) to aid with structuring. RWOV is based on finding the positional relationship between the most relevant words to predicting the class of a text. This facilitates machine learning algorithms to use the interaction of not just keywords but positional dependencies (e.g. a relevant word occurs 5 relevant words before some term of interest). As a proof-of-concept, we attempted to classify the hormone receptor status of breast cancer patients treated at the University of Kansas Medical Center, comparing RWOV to other methods using the F1 score and AUC. RWOV performed as well as, or better than other methods in all but one case. For F1 score, RWOV had a clear edge on most tasks. AUC tended to be closer, but for HER2, RWOV was significantly better for most comparisons. These results suggest RWOV should be further developed for EHR-related NLP.

## Introduction

Since 2015, the federal government has required most healthcare providers in the United States to use electronic medical records (EMR), or suffer penalties to Medicaid and Medicare reimbursement levels^1^. Incentives were also provided to create and use electronic health records (EHR), which are more comprehensive, although the terms are frequently used interchangeably. Many healthcare providers transitioned to EHR well in advance, and consequently, many hospitals now have years of patient records stored in a fashion that is relatively easy to search. In the era of big data, researchers quickly saw the potential to utilize EHR to advance their work in ways that were previously challenging^2–6^. For example, retrospective cohorts for exposures captured in patient records might relatively easily be assembled to study the impact of things like smoking or BMI on outcomes for diseases that are commonly treated at hospitals, such as cancer^5^. EHR have also greatly simplified the process of recruiting subjects for prospective studies and clinical trials^6^. However, the EHR was created for healthcare, not research, and the structure of the data leads to challenges in the research setting.

The biggest challenge in the use of EHR comes from extensive reliance on unstructured data. Healthcare providers typically use freeform notes to capture important information when interacting with patients. Certain types of reports, such as pathology reports, are also entered as free text. Often, some of the most important information is stored in these fields, such as patient descriptions of symptoms, or clinicians’ observation of relevant signs of disease. Due to the volume of data generated by hospitals and other healthcare facilities, collecting data from free text is an arduous process to perform manually. Yet, particularly for clinical trials, this is currently the only available option at many institutions. Given the importance of clinical trials to drug development for a range of conditions, there is a critical need for methods that can automate and improve the efficiency of this process. This led many researchers to propose the application of natural language processing (NLP) techniques to these data^7–12^. NLP is not a specific method but rather a collection of approaches that involve extracting information from language as it is naturally spoken or written. Increasingly, NLP efforts have been focused on EHR to enable researchers to capitalize on its valuable information.

Many NLP approaches are based on two main phases: data structuring and machine learning. In the data structuring phase, an algorithm attempts to impose some sort of regular structure on the data. In the machine learning phase, the newly structured data are used to learn some characteristic of the data (e.g. hormone receptor status for breast cancer patients). The challenge of the data structuring phase is to find some way to impose structure on data that allows meaningful information to be extracted. There is typically a great deal of information in the arrangement of natural language, but it also typically varies a great deal in its arrangement from instance to instance. Therefore, it is difficult to reduce natural language to a structure that works well in most instances.

The field of NLP, as it is applied to EHR, is still developing, and there is a need for methods that are designed specifically to capitalize on the context of EHR. It is our hypothesis that methods specifically designed to exploit the nature of free text in the EHR should exhibit better performance than more general approaches. We note that while some fields may be unstructured, healthcare providers often state things in similar ways, for example estrogen receptor (ER) status may be given as any of the following: “estrogen receptor positive”, “ER+”, “positive for ER”, “positive staining for ER”, “ER: 30%”, and still other variations. Nevertheless, the vocabulary around ER status is relatively constrained. Additionally, the order of text in a medical record is frequently important, as multiple results might be included near each other in the text (e.g. “positive for ER, negative for PR”). As part of our own work to support research at the University of Kansas Cancer Center using the EHR^13^, we are investigating methods for using NLP to extract information from free text fields. In this article, we propose an approach we call Relevant Word Order Vectorization (RWOV). RWOV examines a text for its association to some target class (e.g. ER status). It identifies the most common terms that occur in relation to this target and then scores a text to show the relative position of these most relevant terms to the target. This differs from other methods by considering only the relative position of specific terms, which are assumed to be predictive, to a target term, with extreme flexibility in the true distance between those terms.

## Methods

All work was performed in accordance with relevant guidelines and regulations under a protocol approved by the University of Kansas Medical Center Institutional Review Board, which included a waiver of informed consent.

### Relevant word order vectorization (RWOV)

In this work, we focus on the data structuring part of the NLP problem, thus we will pair our vectorization-based approach with a couple of different machine learning algorithms to compare its performance to existing methods. The basis of our proposed method is to determine the structure of the most relevant words as they relate to predicting the class of text. Therefore, we call this approach Relevant Word Order Vectorization (RWOV). Pseudocode for the basic approach is shown in Algorithm 1.

**Algorithm 1:**
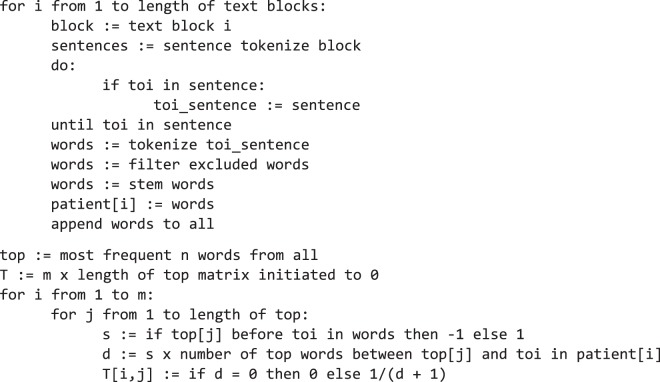
.

In Algorithm 1, *tokenize* refers to breaking the input apart, first into sentences, then into words or symbols. The phrase *excluded words* refers to common “stop words” as they are often called, which for our purposes were the following: *the*, *are*, *of*, *as*, *is*, *and*, *or*, *report*, *pathology*. These were deemed not to be useful for our purposes, although this list could almost certainly be added to and refined. The variable *m* refers to the number of observations (i.e. pathology reports about individuals).

The idea behind RWOV is quite simple. RWOV is focused on predicting the class of a term of interest (TOI) from a block of text. Although EHR data are unstructured, nevertheless, there is a relatively concise vocabulary that is used by healthcare professionals when describing patient characteristics. Therefore, we should see the same terms occurring repeatedly in patient medical records. Furthermore, we propose that only a fraction of these terms indicate the meaning in relation to some particular term of interest. Nevertheless, we believe that the relative order of these “most relevant words” is important to the meaning of the text. RWOV creates a matrix, where each row represents a subject and each column a word. The most relevant words are those that co-occur the most frequently with respect to some TOI. We will call these the top words. The value in each cell of the matrix is either 0, or the inverse of the number of top words that occur between the top word represented by the column and the TOI plus 1. The sign of the value indicates if the top word occurs before or after the TOI in the text. Therefore, the value in each cell drops away naturally in a nonlinear fashion from 1 (as close as possible to the TOI) to 0 (does not occur in the block of text).

$$T(i,j)=\{\begin{array}{ll}0,\, & if\,term\,j\,does\,not\,occur\,in\,block\,i\\ \frac{1}{d+1}, & where\,d\,is\,the\,number\,of\,most\,relevant\,terms\,between\,term\,j\,and\,TOI\end{array}$$

### Data

For this study, we used a straightforward collection of data to evaluate the performance of our NLP approach compared to a few other existing approaches. Three datasets containing tumor pathology reports of women with breast cancer who sought treatment at the University of Kansas Medical Center in two recent years were included, one for each of the terms of interest we used in this study. Our goal is to identify the status of three important breast cancer biomarkers from the pathology report free text: estrogen receptor (ER), progesterone receptor (PR), and human epidermal growth factor receptor 2 (HER2). In order to keep the results more interpretable, we limited the datasets to include only those reports that included a determination of hormone receptor status. The number of positive and negative subjects for each hormone receptor are shown in Table 1.

**Table 1:** Counts of subjects with hormone receptors status.

Hormone Receptor	Positive (%)	Negative (%)	Total
Estrogen receptor (ER)	491 (78.1)	138 (21.9)	629
Progesterone receptor (PR)	396 (66.1)	203 (33.9)	599
Human epidermal growth factor 2 (HER2)	42 (18.3)	187 (81.7)	229

The data were labelled using two annotators who received training in reading pathology reports from clinical research coordinators who normally perform this task. Any discrepancies in annotation were then manually resolved (there were no disagreements due to interpretation).

With respect to Algorithm 1, the specific terms of interest (TOI) used in this study were “er”, “pr”, and “her2”. However, we allow the user to set aliases for TOI. In this case the aliases were for “er”: “estrogen” and “estrogen receptor”, for “pr”: “progesterone receptor”, and for “her2”: “her-2” and “her/”.

Note that in most cases, there were numerous biomarkers listed in a single block of text. For example, a report might list the following: “tumor cells are positive for CK7 and MOC31, negative for CK5/6, TTF-1, CK20, ER and PR. Her2 immunostain is positive. Ki-67 is reported 85%”. In most cases there were between 3–10 biomarkers delineated, adding to the challenge of correct classification.

### Analysis

Class imbalance is likely to be a factor when assessing performance with these types of data. Hormone receptor status is unbalanced in the population. Furthermore, depending on the study, researchers may be interested in one class or the other of the subjects. Complicating this, is the fact that some performance metrics, such as accuracy, can give the impression of good performance even when a method is unable to accurately predict the class of interest. Therefore, we will break down the performance by class (and train separate models to predict each class), and provide F1 and AUC as our major performance metrics, given that accuracy can be misleading in these circumstances. We will not attempt to use sophisticated class balancing approaches in this assessment, so that we can minimize the number of factors that are being considered. F1 is defined as follows:

$$F1=\frac{2\times \frac{TP}{TP+FP}\times \frac{TP}{TP+FN}}{\frac{TP}{TP+FP}+\frac{TP}{TP+FN}}$$

where TP, FP, and FN stand for the number of true positives, false positives, and false negatives respectively. In other words, it is the harmonic mean of the precision and the recall. We will assume that each of our NLP methods can produce a score representing its confidence in the predicted class label of a sample. For this work, we will also assume that a sample can only be one of two classes. Then the AUC is simply the probability that a random sample that is truly of one class is scored higher than a random sample from the other class.

We will compare the performance of our approach to two popular vectorization methods. The first is known as n-grams^9,11^, and the other is called word2vec^14^, combined with either of two machine learning algorithms: support vector machines (SVM), and artificial neural networks (NN). For n-grams, we rely on the implementation in CountVectorizer module of the scikit learn library for python. For word2vec, we used the genism library for python, and chose the skip-gram model. For SVM, we used the SVC module, and for NN we use MLPClassifier. There are two important stages to consider, with their own associated algorithms: (1) data structuring or vectorization and (2) learning and prediction. At both of these stages, important choices can be made that affect performance. At the first stage, vectorization, there are hyperparameters for all of these approaches. For our approach, the only hyperparameter is the number of top (or most relevant) words to model. This was decided by training and testing on an independent dataset and then this setting was simply used for all the analyses here. This will be the recommended default settings for our method, but likely performance could be improved by using training, validation, and test data. However, we wanted to preserve our sample size in this case. For n-grams, we used a number of different settings to try and determine the effect of considering greater or fewer numbers of words. These were [1,2], [2,2], [1,3], [2,3], and [3,3]. The numbers represent the ranges of the number of words to build n-grams from. Furthermore, the vectors were transformed by IDF^15^. The word2vec approach has more hyperparameters, so we searched a number of parameter sets on the independent dataset to determine those with the best performance. We found settings that are in a relatively common range (size = 100 for dimensionality of the vectors, window = 6 for the maximum distance between a word and the predicted word, negative = 5 for sampling negative words). The neural network structure was determined using a grid search to determine the best structure for the n-grams and word2vec methods. Although the differences in performance were minimal overall, the same structure was found for both these methods. Therefore, we also used this structure for our own method, in order to give those methods any possible advantage. This structure contained three hidden layers using rectified linear units (ReLU) for their activations with 50, 50, and then 100 nodes respectively, and a final sigmoid layer for classification. L2 regularization of the network was also used, and the hyperparameter was determined separately for each method using the independent dataset. Again, better performance could be achieved by tailoring this solution. As noted, we have taken measures to ensure the comparison is as fair as possible. In all comparisons, the exact same training and test data were used to compare all models. All results are presented as the average result over a threefold cross-validation.

## Results

Our results (Table 2) showed that RWOV has consistently high accuracy in detecting ER, PR, and Her2 status compared to other vectorization approaches, using NN as a classifier. Furthermore, this result is true irrespective of which class is being predicted. In terms of class imbalance, the other approaches saw a noticeable decline in performance for the underrepresented class in most cases (i.e. ER−, PR−, and HER2+), particularly in terms of F1 score. This is despite the fact that class weighting was enabled for SVM. In every case, RWOV had the highest F1 score. This is particularly notable for the HER2+ class, which included only 42 subjects that were HER2+.

**Table 2:** Classification performance based on vectorization method.

*Method*	ER+		ER−		PR+		PR−		HER2+		HER2−	
	*F1*	*AUC*	*F1*	*AUC*	*F1*	*AUC*	*F1*	*AUC*	*F1*	*AUC*	*F1*	*AUC*
RWOV-NN	**0**.**95**	**0**.**95**	**0**.**82**	**0**.**96**	**0**.**93**	**0**.**96**	**0**.**86**	0.95	**0**.**68**	**0**.**89**	**0**.**91**	**0**.**86**
RWOV-SVM	0.90	0.88	0.70	0.88	0.76	0.72	0.58	0.71	0.53	0.77	0.84	0.77
SVM(1,2)	0.92	0.94	0.69	0.94	0.91	0.95	0.81	0.95	0.31	0.67	0.87	0.68
SVM(2,2)	0.93	0.94	0.72	0.94	0.91	**0**.**96**	0.82	**0**.**96**	0.37	0.73	0.90	0.74
SVM(1,3)	0.94	0.94	0.73	0.94	0.90	0.95	0.80	0.95	0.32	0.69	0.89	0.70
SVM(2,3)	0.94	0.94	0.75	0.94	0.91	0.95	0.82	0.95	0.40	0.72	**0**.**91**	0.74
SVM(3,3)	0.93	0.92	0.71	0.92	0.90	0.92	0.79	0.92	0.35	0.71	**0**.**91**	0.72
SVM-W2V	0.71	0.64	0.40	0.64	0.67	0.69	0.57	0.69	0.20	0.45	0.60	0.43
NN(1,2)	0.93	**0**.**95**	0.71	0.94	0.90	0.95	0.82	0.95	0.35	0.81	**0**.**91**	0.79
NN(2,2)	0.93	0.93	0.72	0.94	0.92	**0**.**96**	0.81	0.95	0.39	0.80	**0**.**91**	0.80
NN(1,3)	0.94	0.94	0.72	0.95	0.91	0.95	0.81	0.95	0.30	0.79	**0**.**91**	0.79
NN(2,3)	0.93	0.92	0.70	0.93	0.92	0.94	0.80	0.95	0.33	0.78	0.90	0.78
NN(3,3)	0.93	0.92	0.69	0.91	0.89	0.92	0.79	0.92	0.20	0.76	0.90	0.76
NN-W2V	0.85	0.76	0.40	0.75	0.81	0.75	0.63	0.78	0.07	0.43	0.87	0.41

RWOV had the most consistent performance across classification tasks. The best performing method for each metric on each task is shown in bold in the table. In only one case did RWOV-NN not have the best performance (PR− AUC), however it was very close to the top performer.

For both F1 score and AUC, RWOV-NN (i.e., RWOV using neural networks), had the best performance in every task with the exception of AUC for PR− classification. However, even in that case it was very close (0.95 vs 0.96 for SVM with n-grams). In some cases, the other methods had equal performance to RWOV at particular settings. Difference in AUC were tested using the pROC package for the R statistical environment using the method of DeLong, *et al*.^16^ (Table 3). In most cases, there were not significant differences in terms of AUC. Although for HER2+, and most comparisons for HER2−, RWOV-NN had a significantly greater AUC than any other approach.

**Table 3:** P-values for difference in AUC between RWOV-NN and other methods.

Method	ER+	ER−	PR+	PR−	HER2+	HER2−
RWOV-SVM	**1**.**54E-06**	**3**.**64E-07**	**9**.**35E-28**	**1**.**83E-24**	**6**.**09E-04**	**3**.**51E-02**
SVM(1,2)	3.56E-01	1.85E-01	4.34E-01	9.54E-01	**3**.**60E-08**	**2**.**60E-05**
SVM(2,2)	4.75E-01	2.56E-01	8.66E-01	5.32E-01	**4**.**30E-05**	**4**.**29E-03**
SVM(1,3)	5.70E-01	3.08E-01	3.03E-01	8.78E-01	**1**.**62E-07**	**7**.**30E-05**
SVM(2,3)	5.04E-01	2.55E-01	3.16E-01	9.02E-01	**1**.**23E-05**	**1**.**96E-03**
SVM(3,3)	5.52E-02	**1**.**99E-02**	**3**.**96E-03**	**4**.**40E-02**	**3**.**88E-06**	**3**.**98E-04**
SVM-W2V	**2**.**75E-15**	**1**.**26E-16**	**3**.**40E-19**	**7**.**95E-19**	**2**.**12E-07**	**1**.**82E-05**
NN(1,2)	9.43E-01	3.81E-01	2.68E-01	5.94E-01	**2**.**06E-02**	8.48E-02
NN(2,2)	2.99E-01	1.93E-01	8.79E-01	3.90E-01	**1**.**90E-02**	1.65E-01
NN(1,3)	5.83E-01	5.43E-01	3.53E-01	7.23E-01	**3**.**60E-03**	5.34E-02
NN(2,3)	1.33E-01	6.79E-02	2.14E-01	8.44E-01	**3**.**23E-03**	**4**.**08E-02**
NN(3,3)	5.79E-02	**1**.**13E-02**	**4**.**09E-03**	**9**.**24E-02**	**6**.**36E-04**	**1**.**99E-02**
NN-W2V	**1**.**42E-27**	**1**.**91E-27**	**5**.**89E-31**	**2**.**21E-27**	**1**.**60E-09**	**7**.**34E-07**

Significant results are highlighted in bold.

**Figure 1:**
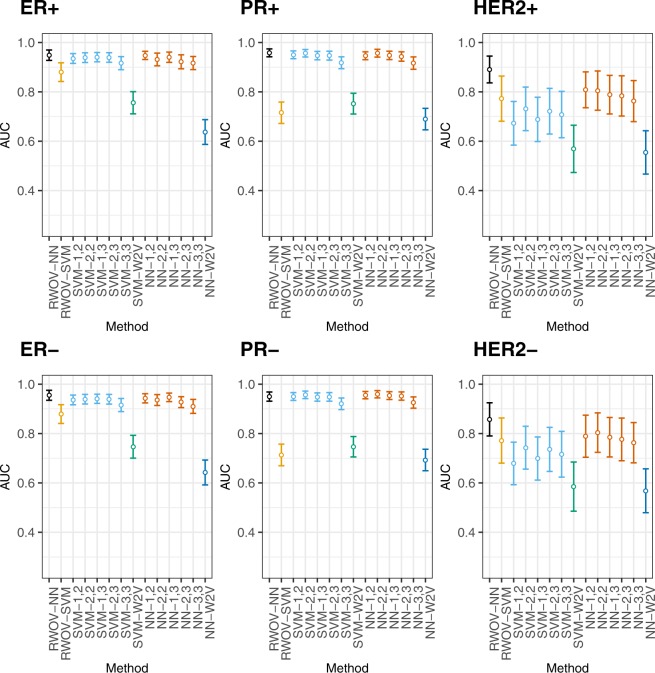
95% confidence intervals for AUC across breast cancer subtypes. Our approach is shown on the left, in black. RWOV-NN has consistently high AUC across the tasks.

**Figure 2:**
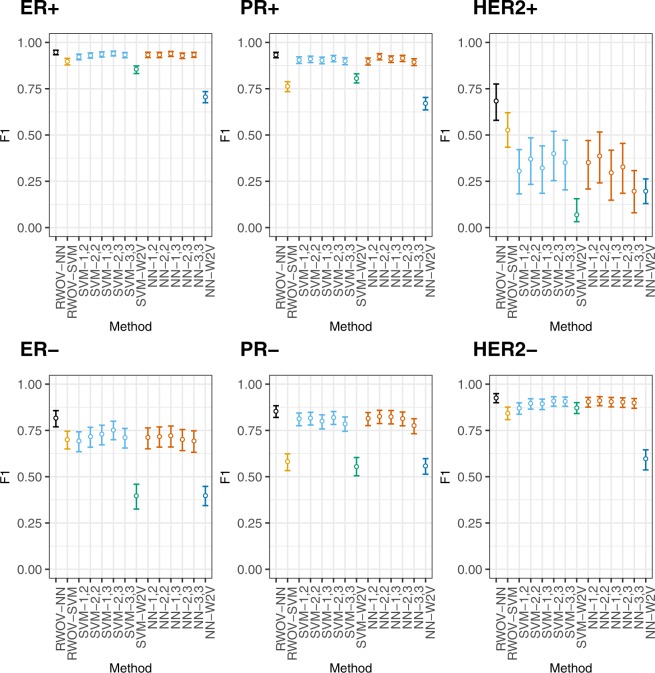
95% confidence intervals for F1 across breast cancer subtypes. Our approach is shown on the left, in black. In every case, RWOV-NN has the highest F1 score across the tasks.

**Figure 3:**
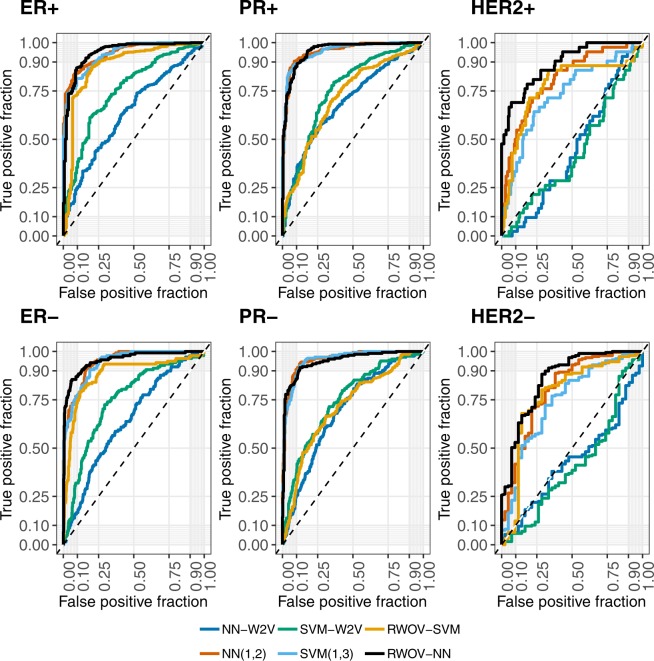
ROC curves for our method, and the best of each of the comparison methods. RWOV-NN shows more clinically useful cut points with low false positives and high true positives are possible consistently across the tasks.

We created 95% bootstrap confidence intervals for all AUCs and F1 scores. For most tasks, RWOV-NN had not only the highest performance but also consistently narrow confidence intervals. These are shown in Figs 1 and 2 respectively. ROC curves are shown in Fig. 3.

From Figs 1 and 2, it can be seen that in every case that there is a significantly superior method, it is RWOV that has the best performance. Figure 3 demonstrates that although there are typically not significant differences in AUC, RWOV-NN typically exhibits the best cut threshold allowing for a high true positive fraction and a low false positive fraction.

The top 30 words for each TOI are shown in Table 4, as well as the mean frequency of their occurrence per observation. Note that the words have been stemmed to reduce them to their common roots or parts, often by truncation, in some cases by substitution with an identifier. For example “tumor” is “tum” and “comma” and “,” are “comm”.

**Table 4:** Top 30 occurring words for each TOI. The mean frequency of occurrence per observation is shown.

Rank	ER Top Words	ER Frequency	PR Top Words	PR Frequency	HER2 Top Words	HER2 Frequency
1	comm	1.16	comm	1.18	comm	0.98
2	pospct	0.96	pospct	1.01	pospct	0.73
3	er	0.71	pr	0.71	her2	0.72
4	tum	0.61	tum	0.58	neg	0.51
5	slash	0.52	slash	0.53	pr	0.44
6	not	0.55	not	0.48	er	0.40
7	receiv	0.44	er	0.42	plu	0.42
8	mark	0.44	posit	0.39	commabef	0.37
9	posit	0.42	between	0.37	tum	0.38
10	ident	0.40	mark	0.37	not	0.37
11	pr	0.39	hour	0.34	stain	0.38
12	outsid	0.35	outsid	0.36	slash	0.30
13	neg	0.34	neg	0.35	mark	0.31
14	prognost	0.33	prognost	0.32	posit	0.31
15	nod	0.30	nod	0.30	on	0.29
16	per	0.28	plu	0.32	outsid	0.29
17	plu	0.29	ident	0.30	in	0.28
18	lymph	0.27	per	0.31	for	0.25
19	for	0.28	lymph	0.28	carcinom	0.29
20	between	0.28	tim	0.27	invas	0.29
21	perform	0.24	ki-67	0.24	cel	0.29
22	necros	0.22	on	0.23	between	0.25
23	sampl	0.22	sampl	0.22	per	0.24
24	ki-67	0.21	negpct	0.21	hour	0.23
25	tim	0.23	1	0.22	nod	0.23
26	hour	0.23	her2	0.22	ki-67	0.24
27	on	0.20	patholog	0.20	prognost	0.24
28	her2	0.19	remov	0.21	stag	0.21
29	follow	0.19	necros	0.20	grad	0.18
30	negpct	0.19	perform	0.21	with	0.19

Words have been stemmed (shortened to common roots/parts).

## Discussion and Conclusions

Electronic healthcare records have enormous promise in facilitating research into improving patient treatment. However, much of the EHR is stored as unstructured data. Therefore, it is time-consuming to extract data from the EHR to pre-screen patients for clinical trials, or perform feasibility analysis for recruitment, because these records must be manually examined. Additionally, useful observational studies could be performed, if it were not for this major limitation. Nevertheless, given that the primary purpose of EHR is to support patient care, it would be inappropriate to change its structure purely to facilitate research. Therefore, it is imperative to develop methods for structuring and learning from these data that can facilitate these goals.

In this study, we have demonstrated that our method, Relevant Word Order Vectorization (RWOV), combined with a neural network, shows great promise in tackling this challenge. On a relevant use case, of identifying the hormone receptor status of breast cancer patients, RWOV showed consistently high accuracy across all three classification tasks. In most cases, it had the highest accuracy of any method examined in this study. Of particular importance, RWOV maintained high accuracy in classes with the poorest representation. This is necessary, because for some studies, it will be necessary to include patients based on these poorly represented classes, and poor accuracy might lead to some subjects being unnecessarily excluded (for example HER2+).

The reason for RWOV’s performance on these tasks seems clear. It depends on a unique vectorization method that determines the most important words for classifying a particular case, in addition to their relative location with reference to a term of interest. This relevant word ordering is well suited to the natural language processing in electronic health records, where the data are semi-structured, due to the repetitive nature of how healthcare providers often enter text. Our algorithm is able to take advantage of this semi-structured data to be a more powerful learner. The relatively poor performance of word2vec, which is a well-respected approach, is likely due to the small sample size. Typically, it depends on larger samples to perform well.

Although RWOV performed at least as well as the other methods for the most common class, it particularly shone in predicting the least common class (ER−, PR−, and HER2+). This was partricularly evident in the case of HER2+. For this dataset, there were relatively few positive observations (just 42 of the 229 total observations), and it has a higher semantic complexity. Although we could not determine a single reason for its performance edge for this problem, in at least some cases it able to recognize a class with a more complex set of dependencies. For example, RWOV-NN and RWOV-SVM correctly classified an observation with a snippet of text like the following (full text longer): “ER: Positive (85%) PR: Positive (80%) HER2: Equivocal (2+) Ki-67: 23% FISH analysis for HER2/neu was performed and was reportedly negative.” The other methods were unable to (with the exception of NN-W2V). For HER2, when the initial test is equivocal, an additional test is performed (FISH analysis), making the classification more complex, especially given the limited sample size. While many approaches focus on finding terms that differentiate text, RWOV focuses on finding positional interactions of terms, which may give it an edge in these scenarios.

This initial approach, although promising, is only the beginning. There are many ways that our method could likely be improved. The learning algorithm was off the shelf, in order to provide proof-of-concept but would likely benefit from more customization, such as tuning the structure of the neural network and applying network optimization methods. Also, we were limited in the amount of data that we could provide, due to the laborious process of hand-labeling examples that is required. Therefore, we think we could improve performance by implementing a semi-supervised approach, in addition to a larger training dataset. We expect that the ideas put forward in this article will stimulate research into other approaches that account for the unique characteristics of EHR. At the same time, RWOV will directly benefit research at the University of Kansas Cancer Center by increasing and improving the data available for projecting the feasibility of studies at this institution. To help other institutions benefit from this approach, RWOV is available for Python in a public code repository at https://github.com/jeffreyat/RWOV.git.
